# Associations of the Dietary Magnesium Intake and Magnesium Depletion Score With Osteoporosis Among American Adults: Data From the National Health and Nutrition Examination Survey

**DOI:** 10.3389/fnut.2022.883264

**Published:** 2022-05-31

**Authors:** Jie Wang, Fei Xing, Ning Sheng, Zhou Xiang

**Affiliations:** Department of Orthopaedics, Orthopaedic Research Institute, West China Hospital, Sichuan University, Chengdu, China

**Keywords:** dietary magnesium intake, magnesium depletion score, bioavailability, osteoporosis, nutrition

## Abstract

**Objectives:**

The study aimed to explore the associations between dietary magnesium (Mg) intake and magnesium depletion score (MDS) among American adults with osteoporosis.

**Methods:**

The continuous data from the National Health and Nutrition Examination Survey 2005–2006, 2007–2008, 2009–2010, 2013–2014, and 2017–2018 were merged to ensure a large and representative sample and a total of 14,566 participants were enrolled for the analysis. The weighted multivariate linear regression model was performed to assess the linear relationship between dietary Mg intake and osteoporosis. Further, the non-linear relationship was also characterized by smooth curve fitting (SCF) and weighted generalized additive model (GAM). In addition, the odds ratios (ORs) and 95% confidence intervals (95% CIs) for associations between the MDS and osteoporosis were assessed by weighted logistic regression models.

**Results:**

After adjusting all covariates, the weighted multivariable linear regression models demonstrated that the dietary Mg intake negatively correlated with osteoporosis, especially in participants aged 55 years or older. In addition, the non-linear relationship characterized by SCF and weighted GAM showed that the dietary Mg intake presented an L-shaped association with osteoporosis among females aged 55 years or older. Moreover, the weighted logistic regression model demonstrated that compared with MDS 0, the OR between MDS ≥3 and osteoporosis was 2.987 (95% CI 1.904, 4.686) in the male-middle intake group. Moreover, compared with MDS 0, the ORs between MDS ≥3 and osteoporosis was 5.666 (95% CI 3.188, 10.069) in the female-low intake group and 1.691 (95% CI 1.394, 2.051) in the female-middle intake group.

**Conclusion:**

The present study indicated that in people with a daily intake of Mg level below the recommended daily intake (RDI), the dietary Mg intake and Mg bioavailability represented by MDS have a negative correlation with osteoporosis. According to the results, the combination of MDS and dietary Mg intake may be more comprehensive and rigorous in screening the population with osteoporosis. Therefore, early monitoring and interventions for osteoporosis may be necessary for those with insufficient dietary Mg intake or high MDS scores.

## Introduction

Osteoporosis is a disease of the skeletal system with degradation of bone tissue microstructure and low bone mineral density (BMD), which usually results in an increased risk of bone fragility and fractures (1). It is estimated that there are 1.5 million osteoporosis-related fractures per year in the US. Fractures can lead to a poor quality of life, a dependent living situation, increased fracture-related mortality, and medical care costs. Furthermore, especially in older adults, hip fractures can be devastating (2). Given the adverse consequences of osteoporosis-related diseases such as fractures, the prevention and management strategies for osteoporosis are of great significance and necessary.

The risk factors that contribute to reduced BMD and osteoporosis are multiple, including genetic, hormonal, environmental, and lifestyle-related factors (3–6). In recent years, various micronutrients, such as magnesium (Mg), have been reported to play an essential role in musculoskeletal diseases. On the one hand, Mg is an essential cofactor for enzymes related to bone matrix synthesis, which promotes bone formation by stimulating osteoblast proliferation. On the other hand, Mg deficiency affects parathyroid hormone (PTH) and Vitamin D levels while promoting inflammatory cytokine secretion and enhancing osteoclast activity (7, 8). However, the results of observational studies about the relationship between dietary Mg intake and osteoporosis were contradictory. Orchard et al. (9) reported that lower dietary Mg intake was related to lower BMD of the hip and whole body, and Ryder et al. (10) found a similar result in white women and men. Moreover, meta-analyses from Farsinejad-Marj et al. (11) and Groenendijk et al. (12) showed that dietary Mg intake was positively correlated with BMD of the femoral neck and total hip. However, no significant associations were found between dietary Mg intake and BMD at other sites. In a study with 2.8 years of follow-up, Kaptoge et al. (13) found that dietary Mg intake was not associated with hip BMD in both men and women, which was supported by Chan et al. (14) and Woo et al. (15).

In addition, previous studies have mainly focused on the effect of dietary Mg intake levels on osteoporosis but ignored the effective bioavailability of dietary Mg. The Mg depletion score (MDS) is a novel scoring tool that integrates several common factors affecting the absorption and excretion of dietary Mg in the US population (8, 16–18). The MDS has been shown to reflect the systemic utilization of the dietary Mg and can identify individuals with relatively low dietary Mg utilization. The higher score represented a lower bioavailability of dietary Mg. Moreover, Fan et al. (19) used the Mg tolerance test to validate MDS as a predictor of real body Mg deficiency in US adults. The results showed that the model containing the MDS alone had the highest area under the receiver operating characteristic curve estimator among models with single predictors, including serum and urine Mg. Thus, MDS may more accurately reflect the real Mg deficiency state of the body. To our knowledge, no previous studies are exploring the relationship between MDS and osteoporosis.

Given the above background, the purpose of the current study was to identify the relationship between dietary Mg intake and osteoporosis and further explore the association between MDS and osteoporosis in US adults.

## Materials and Methods

### Study Population

**Figure 1:**
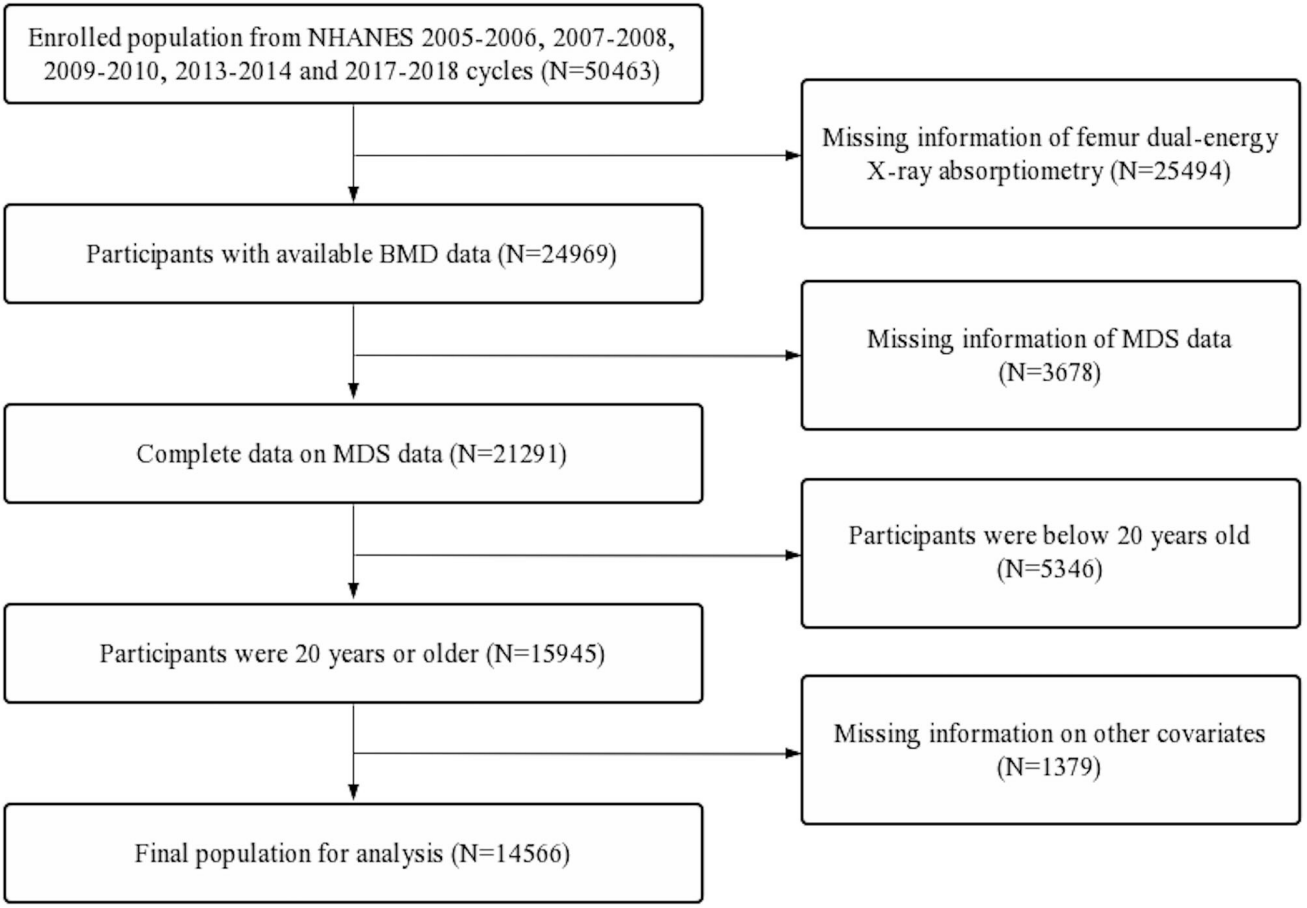
Flow diagram of inclusion criteria and exclusion criteria. NHANES, National Health and Nutrition Examination Survey; MDS, magnesium depletion score; BMD, bone mineral density.

Data used in this study were extracted from the National Health and Nutrition Examination Survey (NHANES). NHANES data were collected from a nationally representative sample of American civilians *via* a multistage probability design. All participants provided written informed consent, and NHANES was approved by the National Center for Health Statistics Ethics Review Board. This study merged the continuous data from NHANES 2005–2006, 2007–2008, 2009–2010, 2013–2014, and 2017–2018 to ensure a large and representative sample. The details of inclusion and exclusion process criteria are shown in Figure 1.

### Dietary Mg Intake and Osteoporosis

Dietary Mg intake data were extracted from two NHANES 24-h recall interviews. The first interview was carried out at the Mobile Examination Center (MEC), and the second was carried out by telephone 3–10 days later. The mean value of the two 24-h recall data was determined as needed dietary Mg intake in the study.

The BMD was evaluated by dual-energy X-ray absorptiometry scans with Hologic QDR-4500A fan-beam densitometers (Hologic, Inc., Bedford, Massachusetts). The assessed femoral regions included total femur, femur neck, trochanter, and intertrochanter. According to the World Health Organization classification criteria, a BMD value in any femoral region lower than −2.5 standard deviations of the reference group can be defined as osteoporosis. The specific thresholds were 0 68, 0.59, 0.49, and 0.78 g/cm^2^ for total femur, femur neck, trochanter, and intertrochanter, respectively (20).

### MDS Calculation

The MDS was calculated by adding up the following 4 points:

1. Current use of diuretics was recorded as 1 point;
2. Current use of proton pump inhibitor (PPI) was recorded as 1 point;
3. Heavy drinker was recorded as 1 point. According to 2015–2020 Dietary guidelines for Americans, the heavy drinkers were defined as >1 drink/d for women and >2 drinks/d for men (http://www.health.gov/DietaryGuidelines);
4. Mildly decreased renal function was recorded as 1 point, and chronic kidney disease (CKD) was recorded as 2 points. According to the Chronic Kidney Disease Epidemiology Collaboration (CKD-EPI) equation (21, 22), the estimated glomerular filtration rate (eGFR) of participants were classified into 3 categories, eGFR ≥90 ml/(min 1.73 m^2^) was defined as normal, 60 mL/(min 1.73 m^2^) ≤ eGFR <90 ml/(min 1.73 m^2^) was defined as mildly decreased renal function, and eGFR <60ml/(min 1.73 m^2^) was defined as CKD.

### Covariates

Based on the previous literature and clinical experience, the selected covariates were obtained as follows:

1. Demographic data: age (<55 years, ≥55 years), sex (male, female), race/ethnicity (Mexican Americans, other Hispanic, non-Hispanic White, non-Hispanic Black, other race), educational level (<9th grade, 9–11th grade, high school, some college, college graduate), marital status (married, widowed, divorced, separated, never married, living with partner), and poverty income ratio (PIR) (<1, 1–3, ≥3);
2. Dietary data: dietary calcium and energy intakes (the mean value of the two 24-h recall data).
3. Examination data: body mass index (BMI) (<25, 25–30, ≥30);
4. Questionnaire data: alcohol consumption (drink/d), smoked at least 100 cigarettes (yes or no), ever use prednisone or cortisone daily (yes or no), moderate or vigorous activity (yes or no).

Comprehensive data: hypertension status (yes or no) and diabetes status (yes, no or borderline). hypertension status was defined according to the following criteria: doctor told you have hypertension, use of hypertension drugs, or mean value of 3 measured diastolic blood pressure ≥90 mmHg or the mean value of 3 measured systolic pressure ≥140 mmHg (The reading with zero is not used to calculate the diastolic average, and if only one blood pressure reading was obtained, that reading is the average). Diabetes was defined according to the following criteria: doctor told you have diabetes, self-reported diabetes for a long time, glycated hemoglobin >6.5%, fasting glucose ≥7.0 mmol/L, random blood glucose ≥11.1 mmol/L, 2-h oral glucose tolerance test blood glucose ≥11.1 mmol/L, and use of diabetes medication or insulin (borderline diabetes = impaired fasting glycaemia or impaired glucose tolerance or prediabetes).

### Statistical Analysis

According to the weight selection criteria of NHANES, sampling weights were used in all analyses. Chi-square test was used to compare the differences of categorical variables between the osteoporosis and non-osteoporosis groups, and for continuous variables, a Student's *t*-test was used. Weighted multivariate linear regression model was performed to assess the linear relationship between the dietary Mg intake and osteoporosis. Subgroup analyses based on sex and age were further performed *via* weighted stratified line regression models. Moreover, the non-linear relationship was characterized by smooth curve fitting (SCF) and weighted generalized additive model (GAM). We also used two-piecewise linear regression models and a recursive algorithm to find the inflection points. Then, the dietary Mg intake were categorized into low, middle, and high groups based on the inflection points of male and female subgroups. In addition, the odds ratios (ORs) and 95% confidence intervals (95% CIs) for associations between the MDS and osteoporosis were assessed by weighted logistic regression models. Subgroup analyses based on sex and the dietary Mg intake levels were further performed *via* weighted stratified logistic regression models. Model 1 was adjusted for no covariates. Model 2 was adjusted for age (if applicable), sex (if applicable), and race. Model 3 was adjusted for all the applicable covariates.

All analyses were performed *via* R software (4.0.3) and EmpowerStats (2.0). A two-sided *p* <0.05 was considered to have statistical significance.

## Results

### Baseline Characteristics of Participants

First, a total of 50,463 participants were extracted. Second, participants with missing femur BMD data (*n* = 25,494) and incomplete MDS data (*n* = 3,678) were excluded. Further, participants below 20 years old (*n* = 5,346) and participants with missing data on other covariates (*n* = 1,379) were also excluded. A total of 14,566 participants were included in the final analysis (Figure 1).

Baseline characteristics of selected participants were compared between osteoporosis and non-osteoporosis groups (Table 1). Among all participants, the prevalence of osteoporosis was 6.9% (*n* = 998). Compared with the non-osteoporosis group, participants in the osteoporosis group tended to have less dietary Mg (263.1 ± 114.3 vs. 304.5 ± 126.5, *P* < 0.001), calcium (854.3 ± 460.8 vs. 963.7 ± 499.6, *P* < 0.001), and energy (1,753.1 ± 709.1 vs. 2,125.9 ± 824.9, *P* < 0.001) intake. After grouping dietary Mg intake by recommended daily intake (RDI, 330.0 mg) and upper limit (UL, 700.0 mg), the percentage of participants whose daily dietary Mg intake below RDI was higher in the osteoporosis group. However, when daily dietary Mg intake was above RDI or UL, the result seemed to be the opposite (*P* < 0.001, Table 1). In addition, the percentage of participants who had a higher MDS, hypertension, diabetes, and ever used prednisone or cortisone daily were significantly higher in the osteoporosis group. Participants in the osteoporosis group were more likely to be female, widowed, older, more emaciated, poorer, smoke more, drink more, have less activity, and have lower educational levels (*P* < 0.050, Table 1).

**Table 1:** Weighted characteristics of the study population.

	**Non-osteoporosis(*N* = 13,568, 93.1%)**	**Osteoporosis (*N* = 998, 6.9%)**	***P*-value**
**MDS (%)**			<0.001
0	40.4	19.7	
1	37.3	39.7	
2	16.5	23.8	
≥3	5.9	16.8	
**Age (years, %)**			<0.001
<55	61.2	13.9	
≥55	38.8	86.1	
**Sex (%)**			<0.001
Male	50.9	17.8	
Female	49.1	82.2	
**Race (%)**			<0.001
Mexican Americans	7.2	3.2	
Other Hispanic	4.4	3.3	
Non-Hispanic White	72.7	83.4	
Non-Hispanic Black	10.0	3.9	
Other race	5.7	6.2	
**BMI (%)**			<0.001
<25	28.8	56.3	
≥25, <30	36.1	28.5	
≥30	35.1	15.2	
**PIR (%)**			<0.001
<1	10.9	11.3	
≥1, <3	33.4	44.9	
≥3	55.7	43.8	
**Educational level (%)**			<0.001
<9th grade	4.6	6.4	
9–11th grade	10.4	12.4	
High school	23.7	29.7	
Some college	30.9	29.2	
College graduate	30.5	22.2	
**Marital status (%)**			<0.001
Married	61.7	47.7	
Widowed	4.8	26.1	
Divorced	11.2	16.2	
Separated	2.2	1.8	
Never married	13.6	5.4	
Living with partner	6.6	2.9	
**Diabetes status (%)**			<0.001
Yes	78.5	71.7	
No	13.5	17.1	
Borderline	8.0	11.2	
**Hypertension status (%)**			<0.001
Yes	66.2	49.4	
No	33.8	50.6	
**Ever use prednisone or cortisone daily (%)**			<0.001
Yes	5.4	8.9	
No	94.6	91.1	
**Smoked at least 100 cigarettes (%)**			0.269
yes	46.5	48.4	
no	53.5	51.6	
**Moderate or vigorous activity (%)**			<0.001
Yes	41.2	56.4	
No	58.8	43.6	
Alcohol consumption (drink/d, mean ± SD)	1.4 ± 3.1	0.7 ± 1.8	<0.001
Magnesium (mg, mean ± SD)	304.5 ± 126.5	263.1 ± 114.3	<0.001
**Magnesium intake level (%)**			<0.001
< RDI	65	78.4	
≥RDI, < UL	33.8	20.7	
≥UL	1.2	0.9	
Calcium (mg, mean ± SD)	963.7 ± 499.6	854.3 ± 460.8	<0.001
Energy (kcal, mean ± SD)	2,125.9 ± 824.9	1,753.1 ± 709.1	<0.001
Total femur BMD (g/cm^2^, mean ± SD)	1.0 ± 0.1	0.7 ± 0.1	<0.001
Femur neck BMD (g/cm^2^, mean ± SD)	0.8 ± 0.1	0.6 ± 0.1	<0.001
Trochanter BMD (g/cm^2^, mean ± SD)	0.7 ± 0.1	0.5 ± 0.1	<0.001
Intertrochanter BMD (g/cm^2^, mean ± SD)	1.2 ± 0.2	0.8 ± 0.1	<0.001

*MDS, magnesium depletion score; BMD, bone mineral density; PIR, poverty income ratio; BMI, body mass index; RDI, recommended daily intake (330.0 mg); UL, upper limit (700.0 mg); SD standard deviation; %, weighted percentage*.

### Associations of Dietary Mg Intake With Osteoporosis

#### Total Analyses

**Figure 2:**
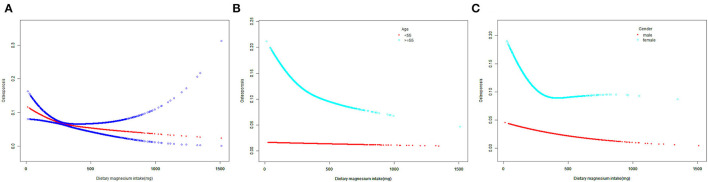
The SCF for associations of MDS with osteoporosis. **(A)** Represents the overall trend. **(B,C)** Represent the substratum trends grouped by age and gender, respectively. Age (if applicable), gender (if applicable), race, BMI, PIR, educational level, marital status, smoked at least 100 cigarettes, hypertension status, diabetes status, ever use prednisone or cortisone daily, moderate or vigorous activity, alcohol consumption, dietary calcium and energy intakes were adjusted. SCF, smooth curve fit; MDS, magnesium depletion score; PIR, poverty income ratio; BMI, body mass index.

The levels of dietary Mg intake showed a negative association with osteoporosis in Model 1. However, after adjusting for confounding factors in Models 2 (age, sex, and race) and 3 (age, sex, race, body mass index [BMI], poverty income ratio [PIR], educational level, marital status, smoked at least 100 cigarettes, hypertension status, diabetes status, ever used prednisone or cortisone daily, moderate or vigorous activity, alcohol consumption, dietary calcium, and energy intakes), the relationship between exposed variables and outcomes remained stable (Table 2). Furthermore, after adjusting for all covariates, the negative associations between dietary Mg intake levels and osteoporosis were also observed in smooth curve fitting (SCF) and weighted generalized additive model (GAM) (Figure 2A).

**Table 2:** Associations of dietary magnesium intake and osteoporosis.

	**Male**	**Female**	**Total**
**Age** **<** **55**			
Model 1 β (95% CI) *P*-value	0.994 (0.993, 0.996)***	1.000 (0.999, 1.001)	0.998 (0.997, 0.999)***
Model 2 β (95% CI) *P-*value	0.994 (0.992, 0.996)***	0.999 (0.998, 1.000)	0.998 (0.997, 0.999)***
Model 3 β (95% CI) *P*-value	1.000 (0.997, 1.003)	1.001 (1.000, 1.002)	1.001 (1.000, 1.002)
**Age** **≥55**			
Model 1 β (95% CI) *P*-value	0.998 (0.997, 0.999)***	0.999 (0.998, 0.999)***	0.999 (0.998, 0.999)***
Model 2 β (95% CI) *P*-value	0.998 (0.997, 0.999)***	0.998 (0.998, 0.999)***	0.998 (0.998, 0.999)***
Model 3 β (95% CI) *P*-value	0.998 (0.997, 0.999)**	0.997 (0.997, 0.998)***	0.998 (0.997, 0.998)***
Total			
Model 1 β (95% CI) *P*-value	0.997 (0.997, 0.998)***	0.999 (0.999, 0.999)***	0.999 (0.998, 0.999)***
Model 2 β (95% CI) *P-*value	0.997 (0.996, 0.998)***	0.999 (0.998, 0.999)***	0.998 (0.998, 0.999)***
Model 3 β (95% CI) *P*-value	0.999 (0.998, 1.000)*	0.998 (0.998, 0.999)***	0.998 (0.998, 0.999)***

*Model 1: no covariates were adjusted*.

*Model 2: age (if applicable), sex (if applicable), and race were adjusted*.

*Model 3: age (if applicable), sex (if applicable), race, BMI, PIR, educational level, marital status, smoked at least 100 cigarettes, hypertension status, diabetes status, ever use prednisone or cortisone daily, moderate or vigorous activity, alcohol consumption, dietary calcium and energy intakes were adjusted*.

PIR, poverty income ratio; BMI, body mass index; *, ** and ***, for P-values <0.05, <0.01 and <0.001, respectively.

#### Subgroup Analyses

In the age below 55 years, dietary Mg intake showed an inverse association with osteoporosis in Models 1 and 2. However, this association did not exist in Model 3 (*P* = 0.080, Table 2). Moreover, when the non-linear relationship was characterized by SCF and weighted GAM, the association between dietary Mg intake and osteoporosis was not significant (Figure 2B). In contrast, at the age of 55 years or older, the relationship between dietary Mg intake and osteoporosis was significantly negative in Models 1, 2, and 3 (Table 2). Further, SCF and weighted GAM presented the negative associations (Figure 2B).

In the male group, dietary Mg intake was negatively correlated with osteoporosis in Models 1, 2, and 3 (Table 2). This negative correlation was further verified by the results of SCF and weighted GAM (Figure 2C). Two-piecewise linear regression model and a recursive algorithm found that the inflection point was 145.5 mg (Table 3). The relationship between dietary Mg intake and osteoporosis in the female group was generally negative in Models 1, 2, and 3 (Table 2). The non-linear relationship between dietary Mg intake and osteoporosis was an L-shaped association (Figure 2C). Further, a two-piecewise linear regression model and a recursive algorithm found that the inflection point was 332.5 mg (Table 3).

**Table 3:** Two-piecewise linear regression models of dietary magnesium intake on osteoporosis.

	**Age <55**	**Age ≥55**	**Total**
**Male**			
Inflection point	222.5	138.0	145.5
< Inflection point	0.982 (0.975 0.988)***	0.973 (0.966, 0.981)***	0.973 (0.967, 0.979)***
> Inflection point	1.003 (1.001, 1.005)*	0.999 (0.997, 1.000)*	0.999 (0.998, 1.000)
Log likelihood ratio	<0.001	<0.001	<0.001
**Female**			
Inflection point	119.0	337.5	332.5
< Inflection point	0.979 (0.969, 0.990)***	0.995 (0.994, 0.996)***	0.996 (0.995, 0.997)***
> Inflection point	1.001 (1.000, 1.003)*	1.002 (1.000, 1.003)**	1.001 (1.000, 1.002)*
Log likelihood ratio	<0.001	<0.001	<0.001

*, ** and ***, for P-values <0.05, <0.01 and <0.001, respectively.

**Figure 3:**
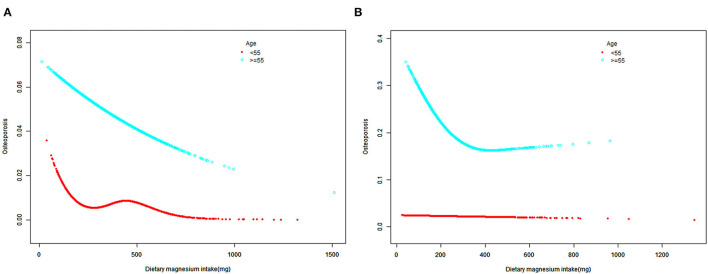
The SCF for associations of MDS with osteoporosis after cross-stratifying by age and sex. **(A)** male; **(B)** female; Race, BMI, PIR, educational level, marital status, smoked at least 100 cigarettes, hypertension status, diabetes status, ever use prednisone or cortisone daily, moderate or vigorous activity, alcohol consumption, dietary calcium and energy intakes were adjusted. SCF, smooth curve fit; MDS, magnesium depletion score; PIR, poverty income ratio; BMI, body mass index.

When the participants were further cross-stratified by age and sex, the negative association between the dietary Mg intake and osteoporosis was mainly presented in males and females aged 55 years or older (Table 2). Meanwhile, SCF and weighted GAM showed that the non-linear relationship between the dietary Mg intake and osteoporosis presented a stable negative correlation in the males aged 55 years or older (Figure 3A). In addition, the non-linear relationship between the dietary Mg intake and osteoporosis presented an L-shape (Figure 3B) in females aged 55 years or older. When daily dietary Mg intake was below the inflection point of 337.5 mg, there was a clear inverse relationship between the dietary Mg intake and osteoporosis. However, when daily dietary Mg intake was more than 337.5 mg, this negative correlation did not exist (Figure 3B, Table 3). In males aged below 55 years, the inverse correlation was only found in Models 1 and 2. However, in females aged below 55 years, subgroup analyses did not show any significant associations between the dietary Mg intake and osteoporosis in Models 1, 2, and 3 (Table 2).

### Associations of MDS With Osteoporosis

#### Total Analyses

The relationship between MDS and osteoporosis generally showed a positive correlation trend. For Model 1, the odds ratios (ORs) between MDS and osteoporosis across scores 1, 2, and ≥3 compared with score 0 were 2.403 (95% confidence interval [CI] 2.194, 2.632), 3.145 (95% CI 2.841, 3.481), and 5.642 (95% CI 5.032, 6.325), respectively. After adjusting for covariates in Model 2, the ORs across scores 1, 2, and ≥3 compared with score 0 were 1.205 (95% CI 1.092, 1.330), 1.075 (95% CI 0.961, 1.202), and 1.623 (95% CI 1.432, 1.839), respectively. Further adjusting for covariates in Model 3, the ORs across scores 1, 2, and ≥3 compared with score 0 were 1.240 (95% CI 1.116, 1.377), 1.161 (95% CI 1.025, 1.316), and 1.785 (95% CI 1.544, 2.064), respectively (Table 4).

**Table 4:** Associations of MDS with osteoporosis.

	**Dietary magnesium intake (mg) <145.5**	**Dietary magnesium intake (mg) ≥145.5, <332.5**	**Dietary magnesium intake (mg) ≥332.5**	**Total**
**Male**				
**Model 1** **β** **(95% CI)** ***P*****-value**				
MDS				
0	1	1	1	1
1	2.094 (1.206, 3.636)**	2.412 (1.796, 3.239)***	2.399 (1.689, 3.406)***	2.342 (1.902, 2.882)***
2	0.511 (0.184, 1.421)	4.455 (3.282, 6.048)***	1.486 (0.928, 2.380)	2.777 (2.194, 3.514)***
≥3	0.188 (0.026, 1.371)	7.139 (5.045, 10.103)***	3.170 (1.789, 5.616)***	4.450 (3.369, 5.877)***
**Model 2** **β** **(95% CI)** ***P*****-value**				
MDS				
0	1	1	1	1
1	2.215 (1.200, 4.091)*	1.002 (0.729, 1.377)	1.414 (0.979, 2.042)	1.206 (0.965, 1.506)
2	0.471 (0.151, 1.466)	1.397 (0.999, 1.955)	0.699 (0.427, 1.142)	1.104 (0.855, 1.425)
≥3	0.161 (0.021, 1.246)	1.822 (1.246, 2.665) **	1.144 (0.629, 2.079)	1.463 (1.083, 1.976)*
**Model 3** **β** **(95% CI)** ***P*****-value**				
MDS				
0	1	1	1	1
1	6.095 (1.594, 23.305)**	1.517 (1.064, 2.161)*	1.471 (0.979, 2.210)	1.480 (1.160, 1.889)**
2	3.682 (0.507, 26.764)	2.936 (1.984, 4.343)***	0.693 (0.398, 1.206)	1.694 (1.269, 2.262)***
≥3	0.329 (0.020, 5.476)	2.987 (1.904, 4.686)***	1.206 (0.610, 2.387)	2.149 (1.521, 3.035)***
**Female**				
**Model 1** **β** **(95% CI)** ***P*****-value**				
MDS				
0	1	1	1	1
1	2.785 (2.031, 3.820)***	2.345 (2.070, 2.656)***	2.188 (1.778, 2.692)***	2.399 (2.168, 2.654)***
2	4.356 (3.066, 6.188)***	3.532 (3.087, 4.040)***	1.852 (1.430, 2.398)***	3.218 (2.875, 3.601)***
≥3	11.898 (8.472, 16.711)***	6.357 (5.477, 7.377)***	1.571 (1.024, 2.409)*	5.909 (5.211, 6.701)***
**Model 2** **β** **(95% CI)** ***P*****-value**				
MDS				
0	1	1	1	1
1	1.493 (1.041, 2.140)*	1.098 (0.958, 1.259)	1.424 (1.138, 1.781)**	1.214 (1.087, 1.356)***
2	1.356 (0.907, 2.028)	1.069 (0.920, 1.242)	0.923 (0.697, 1.222)	1.075 (0.949, 1.217)
≥3	3.276 (2.195, 4.888)***	1.692 (1.435, 1.996)***	0.577 (0.370, 0.899)*	1.677 (1.460, 1.927)***
**Model 3** **β** **(95% CI)** ***P*****-value**				
MDS				
0	1	1	1	1
1	1.683 (1.056, 2.683)*	1.1353 (0.982, 1.313)	1.209 (0.941, 1.554)	1.205 (1.071, 1.356)**
2	1.097 (0.640, 1.882)	1.073 (0.907, 1.268)	1.166 (0.840, 1.620)	1.096 (0.953, 1.261)
≥3	5.666 (3.188, 10.069)***	1.691 (1.394, 2.051)***	0.673 (0.408, 1.109)	1.761 (1.497, 2.071)***
**Total**				
**Model 1** **β** **(95% CI)** ***P*****-value**				
MDS				
0	1	1	1	1
1	2.731 (2.073, 3.598)***	2.351 (2.096, 2.636)***	2.242 (1.876, 2.680)***	2.403 (2.194, 2.632)***
2	3.198 (2.317, 4.414)***	3.675 (3.250, 4.155)***	1.756 (1.401, 2.201)***	3.145 (2.841, 3.481)***
≥3	7.857 (5.773, 10.694)***	6.470 (5.643, 7.418)***	1.967 (1.397, 2.770)***	5.642 (5.032, 6.325)***
**Model 2** **β** **(95% CI)** ***P*****-value**				
MDS				
0	1	1	1	1
1	1.551 (1.144, 2.103)**	1.072 (0.946, 1.215)	1.397 (1.155, 1.691)***	1.205 (1.092, 1.330)***
2	1.115 (0.775, 1.603)	1.119 (0.976, 1.283)	0.841 (0.660, 1.070)	1.075 (0.961, 1.202)
≥3	2.492 (1.741, 3.566)***	1.703 (1.464, 1.980)***	0.711 (0.499, 1.014)	1.623 (1.432, 1.839)***
**Model 3** **β** **(95% CI)** ***P*****-value**				
MDS				
0	1	1	1	1
1	1.768 (1.224, 2.555)**	1.150 (1.007, 1.314)*	1.327 (1.077, 1.634)**	1.240 (1.116, 1.377)***
2	1.263 (0.794, 2.009)	1.222 (1.049, 1.423)**	1.038 (0.791, 1.362)	1.161 (1.025, 1.316)*
≥3	3.607 (2.235, 5.823)***	1.809 (1.518, 2.155)***	0.919 (0.619, 1.365)	1.785 (1.544, 2.064)***

*Model 1: no covariates were adjusted*.

*Model 2: age, sex (if applicable), and race were adjusted*.

*Model 3: age, sex (if applicable), race, BMI, PIR, educational level, marital status, smoked at least 100 cigarettes, hypertension status, diabetes status, ever use prednisone or cortisone daily, moderate or vigorous activity, alcohol consumption, dietary calcium and energy intakes were adjusted*.

MDS, magnesium depletion score; PIR, poverty income ratio; BMI, body mass index; *, ** and ***, for P-values <0.05, <0.01 and <0.001, respectively.

#### Subgroup Analyses

After stratifying the participants by age, the subgroup analyses presented a similar trend to the above. Whether the participants were male or female, the MDS generally showed a positive association with osteoporosis in Model 1. When adjusting for covariates in Models 2 and 3, this trend was partially diminished but still significant. In Model 2 for males, compared with MDS 0, the OR between MDS ≥3 and osteoporosis was 1.463 (95% CI 1.083, 1.976), and in Model 2 for females, the OR was 1.677 (95% CI 1.460, 1.927). In Model 3 for males, compared with MDS 0, the OR between MDS ≥3 and osteoporosis was 2.149 (95% CI 1.521, 3.035), and in Model 3 for females, the OR was 1.761 (95% CI 1.497, 2.071) (Table 4).

Based on the inflection points of 145.5 mg and 332.5 mg, the dietary Mg intake levels were divided into low (<145.5 mg), middle (≥145.5 mg, <332.5 mg), and high (≥332.5 mg) groups. The significant associations between MDS and osteoporosis were mainly found in the low and middle dietary Mg intake groups. In Model 1 of the low intake group, the ORs between MDS and osteoporosis across scores 1, 2, and ≥3 compared with score 0 were 2.731 (95% CI 2.073, 3.598), 3.198 (95% CI 2.317, 4.414), and 7.857 (95% CI 5.773, 10.694), respectively. In Model 1 of the middle intake group, the ORs between MDS and osteoporosis across scores 1, 2, and ≥3 compared with score 0 were 2.351 (95% CI 2.096, 2.636), 3.675 (95% CI 3.250, 4.155), and 6.4697 (95% CI 5.643, 7.418), respectively. In Model 2 of the low intake group, compared with MDS 0, the OR between MDS ≥3 and osteoporosis was 2.492 (95% CI 1.741, 3.566), and in Model 2 of the middle intake group, the OR was 1.703 (95% CI 1.464, 1.980). In Model 3 of the low intake group, compared with MDS 0, the OR between MDS ≥3 and osteoporosis was 3.607 (95% CI 2.235, 5.823), and in Model 3 of the middle intake group, the OR was 1.809 (95% CI 1.518, 2.155) (Table 4).

When the participants were further cross-stratified by sex and dietary Mg intake levels, the male group mainly presented a significantly positive association between MDS and osteoporosis in the middle intake level. On the other hand, the female group mainly presented significantly positive associations between MDS and osteoporosis in both low and middle intake levels. In the male-middle intake group, Models 2 and 3 showed that compared with MDS 0, the ORs between MDS ≥3 and osteoporosis were 1.822 (95% CI 1.246, 2.665) and 2.987 (95% CI 1.904, 4.686), respectively. In the female-low intake group, Models 2 and 3 showed that compared with MDS 0, the ORs between MDS ≥3 and osteoporosis were 3.276 (95% CI 2.195, 4.888) and 5.666 (95% CI 3.188, 10.069), respectively. Moreover, in the female-middle intake group, Models 2 and 3 showed that compared with MDS 0, the ORs between MDS ≥3 and osteoporosis were 1.692 (95% CI 1.435, 1.996) and 1.691 (95% CI 1.394, 2.051), respectively (Table 4).

## Discussion

According to the representative sample of U.S. adults in the National Health and Nutrition Examination Survey (NHANES), we demonstrated that dietary Mg intake levels and osteoporosis were negatively correlated, especially in participants aged 55 years or older. This result suggests that adequate dietary Mg intake may be a factor that prevents osteoporosis in older adults. In addition, we proved that MDS generally presented a significantly positive relationship with osteoporosis. The results of subgroup analyses showed that for males, the positive association mainly presented in the middle Mg intake group. For females, the positive associations mainly presented in both low and middle Mg intake groups. To our knowledge, this is the first study to combine the dietary Mg intake with the bioavailability of Mg and comprehensively explore the association between MDS and osteoporosis.

As we all know, Mg is an essential mineral involved in bone metabolism (7, 8). However, more than half of US adults do not meet the estimated average requirement (EAR) or RDI of daily Mg intake (23, 24). In the present study, the mean daily dietary Mg intake levels were 304.5164 mg ± 126.4613 mg in males and 263.1411 mg ± 114.2658 mg in females (Table 1) and were also far below EAR and RDI. Dietary Mg mainly comes from green vegetables, unpolished grains, nuts, and shellfish, and Mg content in food is easily lost during cooking and refining processes. Moreover, western diets are often rich in refined foods while deficient in green vegetables, and this may help explain the widespread dietary Mg deficiency in the US (25). Given the prevalence of dietary Mg deficiency in the US and the essential role of dietary Mg plays in the bone, comprehensively evaluating the relationship between dietary Mg intake levels and osteoporosis in the US is necessary. The present study showed a negative effect of dietary Mg intake deficiency on the prevention of osteoporosis, which was supported by the results of several previous literature (9–12). To better characterize the relationship in detail, we also performed a two-piecewise linear regression model and a recursive algorithm to find the inflection points of dietary Mg intake in the subgroups with a significant relationship. In the participants aged 55 years or older, the inflection points were 138.0 mg for males and 337.5 mg for females (Table 3). From the perspective of preventing osteoporosis, we recommend that the subpopulation whose daily dietary Mg intake was below the inflection point should be more alert to osteoporosis.

When exploring the effect of dietary Mg on osteoporosis, the bioavailability of dietary Mg should also not be ignored. Clinically, serum Mg is routinely used to diagnose systemic Mg deficiency. However, Mg in the human body is mainly stored in bones and soft tissue (26). Serum Mg only accounts for 0.3% of the whole body Mg content (27). Previous studies have shown that serum Mg was not sensitive to a decline in the actual Mg stores of the body. In addition, individuals with normal serum Mg may have Mg deficiency and respond to Mg supplementation (28, 29). Compared with other methods, the Mg tolerance test (MTT) is more accurate in evaluating the systemic Mg status (30). The test requires measuring the Mg level in 24 h urine and then performing an intravenous drip of Mg for 4 h and collecting the second 24 h urine. However, the relatively complex process limits its widespread application (19, 30, 31). Therefore, an accurate, simple, and convenient tool that can be widely used to evaluate the bioavailability of dietary Mg is urgently needed. The absorption and excretion of dietary Mg can be affected by several factors. For instance, alcohol abuse can lead to a rapidly increased excretion of urinary Mg (18, 32). Proton pump inhibitors (PPIs) can reduce intestinal Mg absorption by interfering with the activity of epithelial Mg^2+^ transient receptor potential channel subfamily M, member 6, and the use of thiazide and loop diuretics were also proved to result in a Mg deficiency (16). In addition, plasma Mg homeostasis is primarily regulated by the kidneys, which are responsible for over 80% of the ultrafiltration and reabsorption of plasma Mg (33). Thus, renal insufficiency of various causes can also affect Mg reabsorption (8). Fan et al. (19) found that Mg levels as determined by MTT had a significant correlation with estimated glomerular filtration rate (eGFR), and Mg deficiency is positively correlated with the severity of renal insufficiency. As a reflection of the Mg bioavailability, MDS combined all the above factors. Meanwhile, the higher the MDS, the poorer the bioavailability of dietary Mg. The accuracy of MDS as a predictive tool for systemic Mg deficiency has been validated by MTT (19).

This study comprehensively assessed the relationship between MDS and osteoporosis. In subgroup analyses based on dietary Mg intake levels, this study found that MDS positively correlated with osteoporosis in the low and middle dietary Mg intake levels. Furthermore, when adding sex to the stratification factor, the positive associations remain stable in the male-middle intake, female-low, and female-middle intake groups. However, this relationship was not significant in the male and female-high intake groups. Similarly, the percentage of participants whose daily dietary Mg intake below RDI was higher in the osteoporosis group, but when daily dietary Mg intake was above RDI or UL, the result seemed to be the opposite. Given that this study used a daily dietary Mg intake of 337.5 mg as the cut-off point for the middle and high groups, which were close to the daily RDI of a US adult (23, 34), these findings may indicate the following three points: (1) in the case of inadequate dietary Mg intake, insufficient Mg bioavailability by the body may further increase osteoporosis, especially in people whose daily dietary Mg intake is below the RDI; (2) the adverse effect of insufficient Mg bioavailability on bone appeared to be partially eliminated by the adequate intake of dietary Mg; and (3) from the perspective of preventing osteoporosis, when dietary Mg intake is below the RDI, increasing dietary Mg intake is beneficial. However, when the dietary Mg intake exceeds the RDI or even reaches the UL, the effect on preventing osteoporosis may deserve further exploration.

There are several strengths in our study. First, we used a large nationally representative database collected *via* standardized protocols to minimize possible bias. Secondly, we adequately controlled for confounders and performed subgroup analyses according to different stratification variables to make the study results more rigorous. In addition, our study has some potential limitations. First, since the present study was an across-sectional analysis, the evidence for a causal relationship may not be sufficient. In the future, more prospective studies need to be performed to confirm the results in the present study. Second, the data collected from the questionnaires and interviews may result in recall bias. Third, although we have adjusted some covariates, other unmeasured confounding factors may also lead to potential bias. Lastly, vitamin D metabolism is dependent on Mg as a cofactor (35), and metabolic balance between PTH and vitamin D has been shown to be closely related to osteoporosis (36). Therefore, dietary Mg deficiency may affect osteoporosis (37) by altering the balance between vitamin D metabolite and PTH. The present study was unable to verify this mechanism due to lack of information on PTH and vitamin D.

## Conclusion

This study indicated that in people with a daily intake of Mg level below the recommended daily intake (RDI), the dietary Mg intake and Mg bioavailability represented by MDS have a negative correlation with osteoporosis. According to the results, the combination of MDS and dietary Mg intake may be more comprehensive and rigorous in screening the osteoporosis population. Therefore, early monitoring and interventions for osteoporosis may be necessary for those with insufficient dietary Mg intake or high MDS scores.

## Data Availability Statement

The original contributions presented in the study are included in the article/supplementary material, further inquiries can be directed to the corresponding author.

## Author Contributions

JW, FX, and NS: conceptualization and investigation. JW: methodology, analysis, and writing—original draft. JW, FX, NS, and ZX: writing—review and editing. All authors contributed to the development of this manuscript and read and approved the final version.

## Funding

This work was supported by the National Natural Science Foundation of China (31870961), the International Cooperation Project of the Science and Technology Department of Sichuan Province (Grant No. 2022YFS0099), and Clinical Research Incubation project of West China Hospital of Sichuan University (2019HXFH041).

## Conflict of Interest

The authors declare that the research was conducted in the absence of any commercial or financial relationships that could be construed as a potential conflict of interest.

## Publisher's Note

All claims expressed in this article are solely those of the authors and do not necessarily represent those of their affiliated organizations, or those of the publisher, the editors and the reviewers. Any product that may be evaluated in this article, or claim that may be made by its manufacturer, is not guaranteed or endorsed by the publisher.
